# Two Concomitant Rare Extraglandular Manifestations of Primary Sjögren’s Syndrome: IgA Nephropathy and Autoimmune Hepatitis

**DOI:** 10.31138/mjr.260123.ina

**Published:** 2024-01-29

**Authors:** Rashmi Roongta, Sonali Dey, Sumantro Mondal, Alakendu Ghosh

**Affiliations:** Department of Clinical Immunology and Rheumatology, Institute of Postgraduate Medical Education and Research, Kolkata, West Bengal, India

**Keywords:** glomerulonephritis, IgA nephropathy, Sjögren’s syndrome, hepatitis, autoimmune

## Abstract

Primary Sjögren’s syndrome (pSS) is a systemic autoimmune disease and can rarely present with multiple extraglandular manifestations. Here we report a case of pSS with concomitant IgA nephropathy and autoimmune hepatitis as the initial manifestations. She presented with polyarthralgia, sicca symptoms and persistent fatigue but was asymptomatic for renal and liver involvement. Autoimmune diseases can have overlapping clinical features and occasionally, manifest nonspecific symptoms leading to delay in diagnosis. It is therefore imperative to thoroughly evaluate any patient of pSS for early recognition of the diverse extraglandular features and initiate prompt treatment to improve outcome.

## INTRODUCTION

Primary Sjögren’s syndrome (pSS) is a systemic autoimmune disease that primarily causes exocrine gland dysfunction. Extraglandular involvement is more likely to occur in patients with positive serologies (serum anti-SSA, anti-SSB antibodies and rheumatoid factor), hypergammaglobulinemia, cryoglobulinemia, and hypocomplementemia. Only approximately 15% of patients with pSS develop severe extraglandular disease.^1^ Renal involvement can be heterogenous varying from renal tubular acidosis to Tubulointerstitial Nephritis (TIN) or glomerulonephritis (GN). IgA nephropathy (IgAN) is the most common primary GN worldwide but its association with pSS has been scarcely reported in literature. It results in kidney failure in 40% of patients within 20 years after diagnosis.^2^ Autoimmune hepatitis (AIH) can be asymptomatic or present with fulminant hepatitis and has a 5-year mortality above 50% if left untreated.^3^ Hence, timely diagnosis and treatment of extraglandular manifestations of pSS are of utmost importance. We report a case of pSS with concomitant IgAN and AIH as the first manifestations and to the best of our knowledge, only one such case has been reported in literature.^4^

## CASE PRESENTATION

A previously well 30-year-old married woman presented to us with polyarthralgia, dry eyes, and persistent fatigue for the past 3 months. General survey and systemic examination were unremarkable. Schirmer test was positive (right eye 5mm, left eye 3mm).

**Figure 1A–B.**
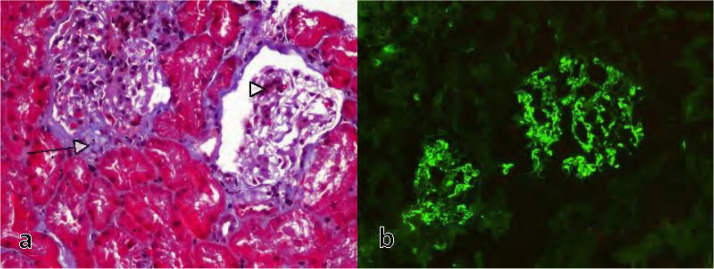
Renal biopsy showed mesangial hypercellularity in all viable glomeruli and immunofluorescence demonstrated mesangial deposits of IgA (2+).

Her initial workup revealed normal complete blood count, thyroid stimulating hormone, erythrocyte sedimentation rate, and C- reactive protein. The basic metabolic panel was normal except for elevated alkaline phosphatase, ALP [171 U/L, normal (44 - 147)], alanine aminotransferase, ALT [243U/L, normal (7–40)] and aspartate aminotransferase, AST [218 U/L, normal (7–40)]. The viral hepatitis panel was negative and serum iron panel and ceruloplasmin were normal. Ultrasonography of the abdomen was normal. Antinuclear antibody (ANA) and anti-smooth muscle antibody (ASMA) were strongly positive at 1:160 and 1: 100 dilution respectively. Anti SS-A and anti SS-B antibodies were positive at high titres of 213U/ml (<20 U/ml) and 40U/ml(<20U/ml) respectively. Serum Immunoglobulin G (IgG 2560mg/dl, reference 700–1700mg/dl) and A (IgA 642mg/dl, reference 70–350mg/dl) were elevated. Complement and cryoglobulin levels were normal and rheumatoid factor was negative. Urine microscopy showed 6–7 red blood cells per high power field (RBCs/hpf) and 24-hour urinary protein excretion was 1.5gm/dl. Labial salivary gland biopsy (Focus score 1) confirmed the diagnosis of pSS. Renal biopsy showed mesangial hypercellularity in all viable glomeruli and immunofluorescence demonstrated mesangial deposits of IgA (2+) (**Figure 1A–B**). The finding was consistent with IgAN (Oxford Classification Score M1E0S1TO-C0).^5^ Liver biopsy was done, which showed chronic lymphoplasmacytic portal inflammatory infiltrates without evidence of steatosis or periportal fibrosis. According to International Autoimmune Hepatitis Group diagnostic criteria (IAIHG),^6^ her score prior to corticosteroid treatment was 17 points and she fulfilled the criteria for definite AIH. The total score was as follows: female (2), reduced ALP/AST (or ALP/ALT) (2), elevated γ-globulin and IgG above normal (1), positive ANA, ASMA (3), negative viral markers (3), no history of utilisation of hepatotoxic drugs and abuse of alcohol (3), presence of plasma cells in histopathology (1) and associated Sjogren’s syndrome (2). The final diagnosis was pSS with IgAN and AIH.

Oral prednisolone 0.5mg/kg/day was initiated in view of IgAN with proteinuria of 1.5gm/dl and AIH. She also received hydroxychloroquine (HCQ) 200mg, oral ramipril 5mg and eye lubricant for ocular dryness. After 4 weeks of prednisolone, aminotransferase levels began to decline. Azathioprine was initiated for AIH as second immunosuppressant. The patient was followed up for one year and there was improvement of the clinical manifestations. Her 24-hour protein excretion reduced to 410mg/dl. Liver enzymes and immunoglobulins also normalised.

The clinical course of the patient is shown in **Table 1**.

**Table 1.** Timeline of the disease and treatment.

**Timeline**	**Symptoms**	**ALP (normal 44–147U/L)**	**ALT/AST (normal <40U/L)**	**Urine microscopy (RBCs/hpf)**	**24-hour protein excretion (gm/dl)**	**Serum creatinine (normal 0.6–1.1mg/dl)**	**Serum IgG (normal 700–1700mg/dl)/IgA (normal 70–350mg/dl)**	**Treatment**	**Outcome**
At diagnosis	Sicca symptoms, fatigue, polyarthralgia	171	243/218	6–7/hpf, albumin 2+	1.5	0.5	2560/642	Prednisolone 0.5mg/kg/day, HCQ 200mg, oral ramipril 5mg, eye lubricant	
After 4-weeks	Fatigue	156	106/94	4–5/hpf, albumin 1+	1.2	0.6	2160/456	Prednisolone was tapered to 0.45mg/kg/day, Azathioprine 50mg added, others continued	Improved
After 1-year	Asymptomatic	120	34/26	normal	0.41	0.5	1200/288	Prednisolone 5mg/day, others as before	Improved and stable

ALP: Alkaline phosphatase; ALT: Alanine aminotransferase; Aspartate aminotransferase: AST; Ig: Immunoglobulin; Red blood cells per high power field: RBCs/hpf; HCQ: Hydroxychloroquine.

## DISCUSSION

The worldwide prevalence of kidney disease in pSS ranged from 1 to 33% depending on the ethnicity and diagnostic criteria. TIN is the typical histological variant in renal biopsy followed by GN which occurs as a late complication of pSS; is relatively rare and has a worse prognosis.^7^ Though IgAN is the most common primary GN worldwide but is rarely reported in pSS. Galactose-deficient IgA1 (Gd-IgA1) and related IgA/IgG containing circulating immune complexes (CIC) were identified as the key drivers in the pathogenesis of secondary IgAN similar to primary IgAN.^8^ Our patient had elevated IgA levels and the pathogenesis of IgAN in pSS might be attributed to the deposition of IgA-containing CIC. A higher prevalence of hypergammaglobulinemia and anti-SSA / anti-SSB antibodies were reported in patients with renal involvement compared to those without renal involvement^9^ and similar findings were seen in our patient.

AIH is characterised histologically by interface hepatitis, elevated liver enzymes and the presence of hypergammaglobulinemia and autoantibodies. It can be classified as type 1 or type 2 depending on the antibodies present.

The prevalence of AIH in pSS ranged from 1% to 4%.^10^ It can be diagnosed based on the IAIHG diagnostic criteria^5^ or the simplified criteria proposed in 2008.^3^ Though liver biopsy was not conclusive in our patient, she fulfilled the IAIHG diagnostic criteria for definite AIH. Moreover, following treatment with corticosteroid there was a dramatic reduction of the liver enzymes. In pSS, HLA DRB1-0301 allele is strongly associated with the Interferon signature and with anti-SSA/anti-SSB production.^11^ Similarly, genetic predisposition to AIH in adults is associated with possession of DRB1*03, -04 alleles.^12^ These basic findings suggest that pSS, IgAN and AIH are attributable to common aetiopathogenesis.

To the best of our knowledge, this is the second case of pSS with concomitant IgAN and AIH.

The first case was a 46-year-old lady who had presented with a four-month history of sicca symptoms with recurrent macroscopic haematuria for one month. She was diagnosed as pSS based on positive anti-SSA and anti-SSB, positive Schirmer’s test and histopathological finding of labial salivary gland biopsy. On evaluation, her urinalysis revealed haematuria, 24-hour urinary protein level was normal and renal biopsy was suggestive of IgAN. She also had elevated transaminase level and strong positive ASMA. She was treated with oral methylprednisolone with gradual tapering and methotrexate was initiated after normalisation of liver function test. She remained well after six months of follow-up.^6^

There are currently no guidelines for the treatment of secondary IgAN. pSS being an immune-mediated disease, immunosuppressants could have a potential benefit in halting the progression of IgAN to kidney failure. As per the American Association of the Study of Liver Disease (AASLD) guidelines glucocorticoid and azathioprine are recommended for the treatment of AIH. Our case demonstrated that prednisolone and azathioprine rapidly decreased transaminase levels and resolved AIH. In addition, oral ramipril and prednisolone resulted in the reduction of proteinuria.

## CONCLUSION

This case highlights the wide array of extraglandular manifestations in pSS. The occurrence of concomitant IgAN and AIH requires early diagnosis and prompt treatment to prevent long-term complications. Further studies are warranted for understanding the pathogenesis of extraglandular involvement in Sjogren’s syndrome to optimise therapeutic strategies.
